# Diphtheria

**Published:** 1863-02

**Authors:** 


*Diphtheria.*—Dr. ISAAC WINANS recommends in Diphtheria: After evacuating the bowels with some mild laxative, the following course: ℞—Tinct. Ferri Muriat. ʒ iij; Acid. Hydrochlor. ℥ j; Aq. Destill. ℥ ss. M. S. Apply to the fauces with a brush or bit of sponge once in six or eight hours until the diphtherious deposit is removed. ℞—Take of the above mixture ʒ j; Aq. Fontan. ℥ ij. M. L. Take a teaspoonful every two hours. If there is debility, add Sulph. Quinine gr. j every second portion. ℞—Chlorat. Potash. ʒ j; Aq. Font. ℥ iv. M. S. Take a teaspoonful every two hours, alternating with the above.

Apply Spirits of Turpentine to the throat externally.

Prof. E. N. CHAPMAN, of the Bellevue College Hospital, ignores about all local treatment in Diphtheria—relying solely upon constitutional measures. Quinine and Brandy are the central means resorted to. We notice also that Dr. MINER, editor of the Buffalo *Journal*, and Surgeon of the hospital in that city, gives it as his opinion that all local applications are useless if not positively injurious. He advises little or no interference, but if indicated, stimulants, food, beef essence, milk punch and support generally, in any available form. Has generally prescribed Dover's Powder and Quinine, but has never been able to see any difference if the quinine is omitted. Prescribes Chlorate of Potash, "mostly from the certainty that, at least, it will do no harm," and that it probably acts on the urinary organs. Chlorinated soda removes the fetor. Dr. Miner remarks that diphtheritic croup often resembles membranous croup so closely that we have no means of differential diagnosis. It often complicates many of the eruptive fevers. In all cases the general plan of treatment remains the same, although the gravity of the disease is greatly augmented.

